# A Case of Congenital Hypothyroidism in Cats: Diagnostic Challenges and Therapeutic Outcomes

**DOI:** 10.1002/vms3.70949

**Published:** 2026-04-30

**Authors:** Morteza Ezati Kakalar, Farkhondeh Fahim Dezhban, Amin Zamani, Sina Sabour Razlighi

**Affiliations:** ^1^ Department of Small Animal Internal Medicine, Faculty of Veterinary Medicine University of Tehran Tehran Iran; ^2^ Department of Radiology and Surgery, Faculty of Veterinary Medicine University of Tehran Tehran Iran; ^3^ Department of Veterinary Medicine Karaj Branch, Islamic Azad University Karaj Iran; ^4^ Department of Internal Medicine Faculty of Veterinary Medicine University of Garmsar Garmsar Iran

**Keywords:** congenital hypothyroidism, dwarfism, feline, levothyroxine, Persian cat

## Abstract

This report presents a case of congenital hypothyroidism in a 3‐year‐old female Persian cat, a condition that is seldom encountered in feline patients. The cat was presented with chronic renal failure and stunted growth, exhibiting a unique combination of symptoms, including dwarfism, an abnormal skull shape and open growth plates. A complete blood count (CBC) identified moderate anaemia, while the biochemical analysis showed elevated serum creatinine levels and blood urea nitrogen (BUN) levels.

Low serum total thyroxine (TT4) concentrations, both before and after the administration of thyroid‐stimulating hormone (TSH), confirmed the presumptive diagnosis of congenital hypothyroidism. Following this, levothyroxine supplementation was initiated. After 1 week of therapy, the cat displayed decreased creatinine levels and increased activity.

This case highlights that feline hypothyroidism, although rare, should be considered a differential diagnosis in similar cases.

## Graphical abstract

Congenital hypothyroidism in a 3‐year‐old Persian cat was diagnosed via low total thyroxine levels unresponsive to TSH stimulation, alongside clinical signs of dwarfism and renal failure. Levothyroxine therapy significantly improved renal function and activity within 1 week. This rare case highlights the importance of considering hypothyroidism in feline patients with growth abnormalities and renal disease for timely diagnosis and effective treatment.

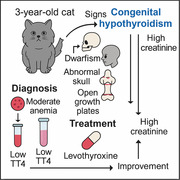

## 1 Introduction

The thyroid is an endocrine gland structure in the ventral cervical region, consisting of two lobes (Ilahi et al. 2025). It secretes three hormones: thyroxine (T4), triiodothyronine (T3) and calcitonin (Ilahi et al. 2025). Calcitonin helps regulate calcium levels in the body by lowering blood calcium (Ilahi et al. 2025). The thyroid gland regulates the body's metabolism and calcium balance (Ilahi et al. 2025). Thyroid hormones increase cellular metabolism and oxygen consumption. The secretion of hormones by the thyroid gland is regulated by the amount of thyroid‐stimulating hormone (TSH) produced by the pituitary gland. The production of TSH, in turn, is regulated by the hypothalamus. Congenital hypothyroidism is sporadic in cats, while it affects approximately one in 4000 infants in humans (Rastogi and LaFranchi 2010). In cats, spontaneous hypothyroidism is more commonly seen in its congenital form than in adult‐onset (Greco 2006). It seems that an autosomal recessive pattern causes inherited forms of hypothyroidism (Altiner and Bilal 2024).

Hypothyroidism may be acquired or congenital (Greco 2006; Altiner and Bilal 2024). Causes of adult‐onset congenital hypothyroidism can include lymphocytic thyroiditis, diffuse follicular hyperplasia and idiopathic causes (Greco 2006). Causes of congenital hypothyroidism include: (1) thyroid dyshormonogenesis, (2) thyroid hypoplasia or aplasia, (3) TSH resistance and (4) thyroiditis.

Kittens affected by congenital hypothyroidism may exhibit short limbs, spines, block‐like trunks and broad, short skulls (Quante et al. 2010). In addition to the hallmark sign of failure to thrive, other clinical signs can include lethargy, impaired mental status, neuromuscular deficits, retention of juvenile hair coats, delayed dental eruption, constipation, bradycardia, hypothermia, sealed eyelids, stenotic ear canals, macroglossia, abdominal distension from the accumulation of myxedematous fluid and bilateral cryptorchidism (Iturriaga et al. 2020).

This case study report outlines the diagnosis, treatment and outcome of a congenital hypothyroidism in a feline patient.

## 2 Case Report

A 3‐year‐old female Persian cat named Ilgar, weighing 2 kg, was presented for a check‐up at a Panah veterinary clinic in Tehran, Iran. The patient exhibited slightly abnormal skull and body contours during the physical examination. Notable characteristics included a minor discrepancy in age‐related stature. All four limbs and the tail were short and stubby, while the head was broad with flattened facial features. The rectal temperature was mildly subnormal at 37.6°C. The patient was neutered 6 months ago, and rapid tests for feline leukaemia virus and feline immunodeficiency virus (FIV) returned negative results.

A complete blood count (CBC) revealed moderate normocytic and normochromic anaemia, with a haematocrit of 21%. The biochemical panel revealed elevated serum creatinine (SCr) levels and blood urea nitrogen (BUN) levels, measuring 5.5 mg/dL and 94.5 mg/dL, respectively (Table 1). In healthy cats, the reference interval for BUN is generally 17–35 mg/dL. For SCr, the reference range is typically 0.8–2.1 mg/dL (Cornell University College of Veterinary Medicine n.d.) (Table 1).

**TABLE 1:** Laboratory results of our patient with congenital hypothyroidism, including biochemical parameters (BUN, creatinine, albumin, phosphate) and haematological indices (WBC, lymphocytes, red blood cells, haemoglobin, PCV), aid in diagnosing primary hypothyroidism and overall patient assessment. The hormonal T4 levels in the TSH stimulation test, both before and after injection, along with clinical signs, help to confirm the diagnosis and guide appropriate treatment for this case.

Biochemistry	Result	Reference interval	CBC	Result		Reference interval
BUN	94.5	17–35 mg/dL	WBC	4.6		5.1–16.2, 10^3^/µL
Creatinine	5.55	0.8–2.1 mg/dL	Lym	16%	0.736 10^3^/µL	9%–56%/0.9–6.0, 10^3^/µL
Albumin	3.43	3.2–4.3 g/dL	Mono	3%	0.138 10^3^/µL	0%–6%/0.0–0.7, 10^3^/µL
P	7.45	2.6–5.5 mg/dL	Eos	9%	0.414 10^3^/µL	1%–15%/0.1–1.8, 10^3^/µL
			Neut	72%	3.312 10^3^/µL	27%–82%/2.3–11.6, 10^3^/µL
**Hormone result**			Band	0%	0.0 10^3^/µL	0%–1%/0.0–0.1, 10^3^/µL
T4 (0) = < 6.00 nmol/L T4 (6) = < 6.00 nmol/L	Before injection 6 h after injection		PLT	Adequate		195—624, 10^3^/µL
According to the following results, primary hypothyroidism is likely (post TSH 6 h less than 1.5 µg/dL confirms primary hypothyroidism; however, in critically ill patients, decreased post TSH T4 occurs).			PCV	19		31%–48%
			RBC	4.61		6.9–10.1, 10^6^/µL
			Hb	6.4		10.9–15.7 g/dL
			MCV	41.21		40–52 fL
			MCH	13.88		13–17 pg
			MCHC	33.68		32–35 g/dL
			RDW	14.9%		10.6%–14.3%

Abbreviations: BUN, blood urine nitrogen; Eos, eosinophils, Hb; haemoglobin, Lym = lymphocytes, MCH = mean cell haemoglobin, MCHC = mean cell haemoglobin concentration; MCV, mean cell volume, Mono, monocytes, Neut, neutrophils; P, phosphate; PCV, packed cell volume; PLT, platelets; RBC. red blood cells; RDW, red blood cell distribution width; WBC, white blood cells.

**FIGURE 1:**
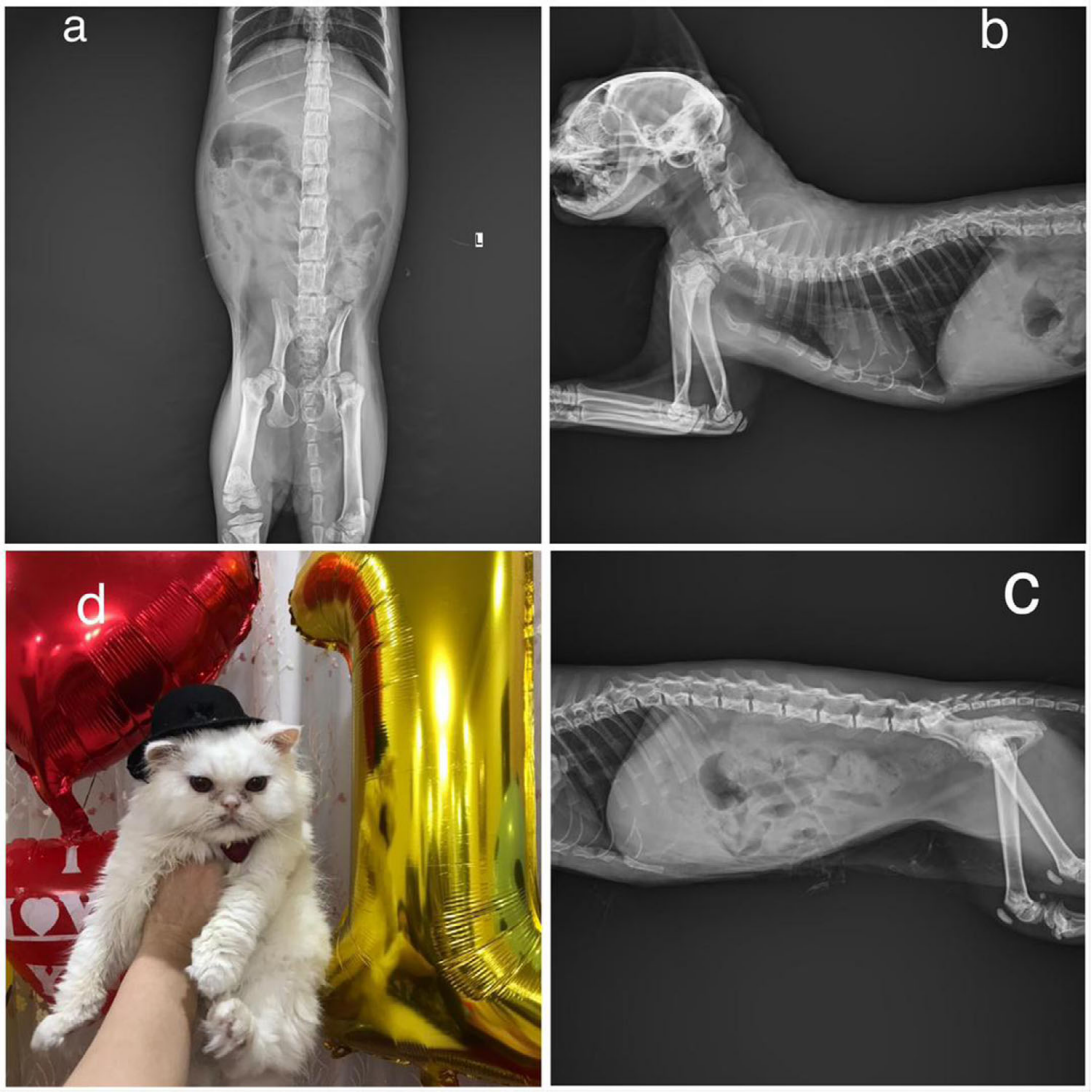
(a) Ventro–dorsal (VD) view of the abdominal and pelvic regions, showcasing notable skeletal abnormalities. Radiographical findings reveal generalized epiphyseal dysplasia affecting both long bones and the vertebral column, characterized by stippling of the epiphyses and irregular epiphyseal margins. The cuboid vertebral bodies are shorter than normal, and the persistent growth plates show delayed mineralization. The coxofemoral joints demonstrate severe shallowness of both acetabula, with the left femoral head nearly dislocated, accompanied by medial rotation of the left hind limb. A significant difference in femur length is noted between the right and left limbs (up to 2 cm), suggesting potential concerns for further evaluation. The stomach is filled with ingesta, and there is clear visibility of the abdominal serosal detail. These findings indicate a diagnosis of mucopolysaccharidosis and raise suspicion of multiple epiphyseal dysplasia, with concerns for dwarfism/achondroplasia. (b) Lateral view of the skull and thoracic spine. The radiograph highlights irregularities in the vertebral bodies and epiphyseal regions, supporting the diagnosis of generalized epiphyseal dysplasia. (c) Lateral view of the thoracic spine and abdomen. This image further reinforces the noted abnormalities in the skeletal structure, including epiphyseal dysplasia and vertebral cuboid shaping, consistent with the initial findings. (d) A photo of our patient.

The most striking feature in this case was mild disproportionate dwarfism. Causes of congenital feline dwarfism can include endocrinopathies such as hypothyroidism, hypopituitarism, juvenile diabetes mellitus and hypoadrenocorticism. Non‐endocrine causes include portosystemic vascular anomalies, renal disease, osteochondrodysplasia, cardiac defects and lysosomal storage diseases. We referred the patient for X‐ray imaging, which surprisingly revealed that most of the growth plates were open (Figure 1a–c).

Mucopolysaccharidosis was also considered as a differential diagnosis, as affected cats may present with growth abnormalities and epiphyseal dysplasia. However, in this case, no skeletal deformities or radiographic evidence of epiphyseal changes were observed, making mucopolysaccharidosis an unlikely diagnosis. The urine sample was analysed for the presence of mucopolysaccharides to aid in the diagnosis of mucopolysaccharidosis, and the result was negative. Due to the economic issues and the high cost of hormonal tests in our country (Islamic Republic of Iran), to reduce the financial burden on the owner, we decided to perform just one test, which is more accurate and more definitive than others such as, serum concentration of tT4, fT4 and TSH. So, we directly proceeded with the TSH stimulation test. In this case, the TSH stimulation test results showed no change between T0 and T1 samples (taken 6 h after intravenous TSH injection), with both serum total thyroxine (TT4) values below normal limits (< 6 nmol/L). The test was performed accordingly. The lack of TT4 response supports primary hypothyroidism, though secondary hypothyroidism cannot be entirely ruled out, given the limitations discussed (Ettinger and Feldman 2024).

Overall, the clinical signs, laboratory results and radiological findings were consistent with a diagnosis of primary congenital hypothyroidism. Due to the presence of growth plates and abnormal body shape, it seems the patient suffered from a congenital form (Figure 1a). In cretinism, the secondary form is very rare (Ettinger and Feldman 2024; Degregori et al. 2020).

## 3 Discussion

This report describes the successful diagnosis and management of a rare case of congenital hypothyroidism in a feline patient. The diagnosis was confirmed using a thyroid dynamic test, which demonstrated a subnormal response. Euthyroid cats typically show a post‐TSH total T4 (TT4) concentration greater than 1.5 times the basal level, with an absolute value exceeding 30 nmol/L. In contrast, our patient exhibited minimal stimulation, with a post‐TSH TT4 measurement below 20 nmol/L, consistent with hypothyroidism.

Although the TSH stimulation test is often considered the gold standard for diagnosing hypothyroidism, elevated TSH combined with decreased total or free T4 levels may also be consistent with a diagnosis. However, it is essential to note that TSH measurement in cats has suboptimal sensitivity (Ettinger and Feldman 2024). Our diagnosis was further supported by the exclusion of mucopolysaccharidosis via negative urine test results and the presence of characteristic clinical features, including open growth plates and mild dwarfism (Figure 1a–d). Given the extreme rarity of secondary cretinism in cats, with only one reported case in the literature (Degregori et al. 2020), primary hypothyroidism was considered the most likely diagnosis. However, economic constraints prevented direct TSH measurement or extended monitoring, which would be required to definitively rule out a secondary form.

Our therapeutic plan involved administering 50 µg of levothyroxine orally every 24 h. The patient showed a remarkably positive response within 1 week of initiating therapy. Creatinine levels decreased from 5.55 mg/dL to 3.33 mg/dL, and the cat's activity levels increased noticeably. Post‐treatment hormonal evaluation revealed a TT4 concentration of 23.71 nmol/L, confirming adequate hormone supplementation (Table 2). This rapid clinical and biochemical improvement underscores the potential for effective management of this condition.

**TABLE 2:** Laboratory results following treatment for congenital hypothyroidism in a feline patient.

Biochemistry	Result	Reference interval	Hormone test	Result	Reference interval
Creatinine	3.33	0.8–2.1 mg/dL	T4	23.71	10–47 nmol/L
			tT4	0.2	0.0–0.38 ng/mL

Congenital hypothyroidism is exceptionally rare in cats, and its vague systemic signs can make recognition challenging, with many affected kittens not surviving to diagnosis (Lim et al. 2014). Thyroid hormones are critical for normal postnatal development of the skeletal and nervous systems (Jacobson and Rochette 2018). They influence bone growth both by stimulating pituitary growth hormone production and by interacting with growth hormone to regulate the sequence of endochondral ossification, which ultimately leads to skeletal closure (Jacobson and Rochette 2018). The common presenting complaint of mental dullness and lethargy in the literature (Bojanic et al. 2011) aligns with the clinical picture in this case.

In conclusion, this case serves as a valuable addition to the sparse literature on feline congenital hypothyroidism. It highlights that despite diagnostic challenges, a combination of clinical signs, dynamic testing and a positive response to therapy can lead to a successful outcome. Cats diagnosed with this condition can lead relatively normal lives with lifelong thyroid hormone supplementation, but should not be used for breeding, as the condition may be inherited as an autosomal recessive trait (Bojanic et al. 2011). By sharing the diagnostic approach, treatment and positive outcome, we hope to enhance the understanding of this condition within the veterinary community.

## Author Contributions

**Morteza Ezati Kakalar**: conceptualization, methodology, data curation, investigation, validation, formal analysis, supervision, visualization, writing – review and editing. **Farkhondeh Fahim Dezhban**: conceptualization, methodology, data curation, investigation, validation, formal analysis, visualization, writing – review and editing. **Amin Zamani**: conceptualization, methodology, data curation, investigation, validation, formal analysis, visualization, writing – original draft, writing – review and editing. **Sina Sabour Razlighi**: conceptualization, methodology, data curation, investigation, validation, formal analysis.

## Funding

The authors have nothing to report.

## Ethics Statement

The authors have nothing to report.

## Conflicts of Interest

The authors declare no conflicts of interest.

## Data Availability

The data supporting this study's findings are available from the corresponding author upon reasonable request.
